# Fructose-Induced Intestinal Microbiota Shift Following Two Types of Short-Term High-Fructose Dietary Phases

**DOI:** 10.3390/nu12113444

**Published:** 2020-11-10

**Authors:** Julia Beisner, Anita Gonzalez-Granda, Maryam Basrai, Antje Damms-Machado, Stephan C. Bischoff

**Affiliations:** Institute of Nutritional Medicine, University of Hohenheim, Fruwirthstr. 12, 70599 Stuttgart, Germany; julia.beisner@uni-hohenheim.de (J.B.); anita.gonzalez@hospitalgonzalez.ec (A.G.-G.); m.basrai@uni-hohenheim.de (M.B.); antje.damms@googlemail.com (A.D.-M.)

**Keywords:** fructose, microbiota, host-microbe interactions, obesity, metabolic syndrome, NAFLD

## Abstract

High consumption of fructose and high-fructose corn syrup is related to the development of obesity-associated metabolic diseases, which have become the most relevant diet-induced diseases. However, the influences of a high-fructose diet on gut microbiota are still largely unknown. We therefore examined the effect of short-term high-fructose consumption on the human intestinal microbiota. Twelve healthy adult women were enrolled in a pilot intervention study. All study participants consecutively followed four different diets, first a low fructose diet (< 10 g/day fructose), then a fruit-rich diet (100 g/day fructose) followed by a low fructose diet (10 g/day fructose) and at last a high-fructose syrup (HFS) supplemented diet (100 g/day fructose). Fecal microbiota was analyzed by 16S rRNA sequencing. A high-fructose fruit diet significantly shifted the human gut microbiota by increasing the abundance of the phylum *Firmicutes*, in which beneficial butyrate producing bacteria such as *Faecalibacterium*, *Anareostipes* and *Erysipelatoclostridium* were elevated, and decreasing the abundance of the phylum *Bacteroidetes* including the genus *Parabacteroides*. An HFS diet induced substantial differences in microbiota composition compared to the fruit-rich diet leading to a lower *Firmicutes* and a higher *Bacteroidetes* abundance as well as reduced abundance of the genus *Ruminococcus*. Compared to a low-fructose diet we observed a decrease of *Faecalibacterium* and *Erysipelatoclostridium* after the HFS diet. Abundance of *Bacteroidetes* positively correlated with plasma cholesterol and LDL level, whereas abundance of *Firmicutes* was negatively correlated. Different formulations of high-fructose diets induce distinct alterations in gut microbiota composition. High-fructose intake by HFS causes a reduction of beneficial butyrate producing bacteria and a gut microbiota profile that may affect unfavorably host lipid metabolism whereas high consumption of fructose from fruit seems to modulate the composition of the gut microbiota in a beneficial way supporting digestive health and counteracting harmful effects of excessive fructose.

## 1. Introduction

The prevalence of overweight and obesity is increasing worldwide. The World Health Organization (WHO) reports that in 2016 39% of adults (> 18 years) were overweight and 13% were obese. The prevalence of overweight children and adolescents has increased dramatically from 4% in 1975 to 18.5% in 2016. Changes in dietary and eating behavior such as consumption of sugar-sweetened beverages and sugar-rich processed food high in fat and refined carbohydrates, the so-called western style diet (WSD) in conjunction with lower levels of physical activity are associated with a rise in obesity [1]. Among the dietetic influences, particularly sucrose- and fructose-rich soft drinks are the most critical factors causing the development of obesity and fatty liver disease.

Fructose naturally appears in fruits and vegetables, but it is also often used as a cheap, refined carbohydrate sweetener in the form of high-fructose-glucose syrup for soft drinks, sweets and highly processed foods. Although the clinical impact of long term high-fructose consumption is still controversial [2], many studies have shown the harmful effects of a high-fructose intake in animal models [3,4,5,6] and humans [7,8,9,10,11]. High-fructose consumption may be associated with obesity [12], metabolic syndrome [13] and non-alcoholic fatty liver disease (NAFLD) [14,15,16,17]. Although the exact mechanism of fructose induced development of NAFLD is still not fully understood [14], it is known that a high-fructose consumption causes epithelial barrier dysfunction by increasing intestinal permeability [18,19,20]. Consequently, endotoxins like lipopolysaccharides (LPS) can translocate through the mucosa into the blood stream leading to metabolic endotoxemia [16,21,22].

Diet is one of the various factors that influences the gut microbiota composition [14,23]. Dietary fructose and glucose, which are prevalent in the Western diet, affect host-gastrointestinal microbe interactions contributing to differences observed in obese and normal-weight intestinal microbiota. However, only a few studies investigated the impact of fructose-rich diets on gut microbiota and the subsequent effects of high-fructose diet-induced effects on metabolic diseases. A high-fructose or a high-sugar consumption has been shown to influence the microbiota composition [24,25] and alters the abundance of *Bacteroidetes* and *Firmicutes* [6,26,27]. In rats fed a fructose-rich diet, the development of metabolic syndrome was correlated with variations of the gut content of specific bacterial genera [28]. Crescenzo et al. [29] reported that a fructose-rich diet promoted alterations in the gut microbiota profile in rats and, moreover, that these alterations were associated with inflammation and metabolic dysregulation in the gut and liver. The substantial link between microbiota dysbiosis and NAFLD has been supported by results from animal studies showing that obesity-related features in fructose-fed rats were reversed by changes in the gut microbiota profile and development of NAFLD was markedly reduced by treatment with antibiotic therapy, prebiotics and selected probiotics [28,29,30]. The fructose-induced microbiota alteration is therefore considered a critical factor contributing to NAFLD progression in animal models and humans and has been associated with the metabolic syndrome [31].

The aim of the present study was to investigate the influence of different types of short-term fructose-rich diets on the human gut microbial signature. Therefore, we analyzed the human microbiota composition of healthy females following consecutively different high-fructose diets, first a fruit diet (100 g/day fructose from fruit and vegetables) and then, after a low-fructose phase, a HFS diet (100 g/day fructose from syrup) and characterized the responses of bacterial communities in the study subjects that underwent the dietary intervention. In a previous study, we found significant changes especially in certain acylcarnitine and lysophosphatidylcholine levels after high-fructose consumption in the same study cohort [32] and therefore also correlated the microbiota abundances with plasma metabolites. Here we report a shift in the bacterial abundances after high-fructose diets.

## 2. Materials and Methods

### 2.1. Study Design

**Figure 1:**
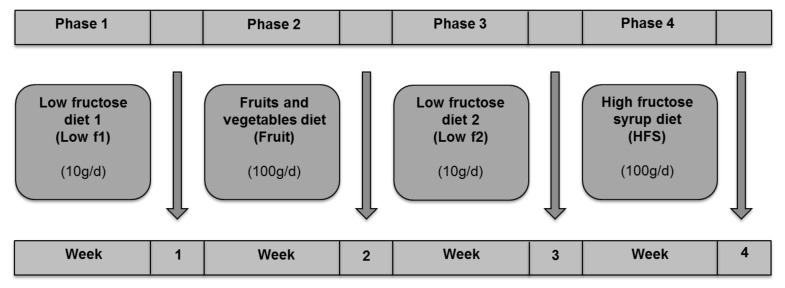
Study design. Study subjects received a low-fructose diet 1 (low f1), a fruits and vegetables diet (fruit), a low fructose diet 2 (low f2) and a high-fructose syrup diet (HFS).

This pilot study is an open-label, single-arm intervention study and included six lean and six obese healthy volunteers, who underwent four different diets. During the first week, study subjects received a low-fructose diet (low f1), which served as control diet. The second week consisted of a high-fructose diet rich in fruits and vegetables (fruit). During the third week subjects again followed a low fructose diet (low f2), which was identical to low f1 and served as wash-out phase. During the fourth week, study subjects received a high-fructose diet supplemented with high-fructose syrup (HFS). The study design is graphically shown in Figure 1.

### 2.2. Selection of Subjects

Twelve healthy female volunteers, aged between 20 and 40 years, were recruited through email distribution from the University of Hohenheim, Germany, over a time period of six weeks. After written informed consent, a medical history, physical examination, blood sample collection and a hydrogen breath test were performed to exclude fructose intolerance before enrollment to ensure eligibility. The subjects had to be non-smokers, and non-pregnant/non-breastfeeding. Women with chronic gastrointestinal diseases who have undergone gastrointestinal surgery (other than appendectomy), women with chronic anemia, chronic hepatic or renal disease, diabetes mellitus or other relevant chronic health disorders or regular medication were not included in the study. Subjects who passed the eligibility criteria were allocated according to their body mass index (BMI) either to the lean (20 < BMI < 25 kg/m^2^, *n* = 6) or to the obese (35 < BMI < 50 kg/m^2^, *n* = 6) group. Table 1 shows the baseline characteristics of the study population. The study was approved by the responsible ethics committee (Landesärztekammer Baden-Württemberg, Stuttgart, Germany; no. 2009–079-f) in 2009, registered at ClinicalTrials.gov (ID: NCT03444233) and carried out in accordance with the Helsinki Declaration (revised version, 1989).

### 2.3. Dietetic Intervention

Before study intervention, all subjects participated in a nutritional training, in which they were informed about the sugar content in foods. Additionally, each subject received an individual nutritional counselling based on an individualized nutritional plan.

The previewed diets were largely isocaloric and isonitrogenous with 30% of total energy intake derived from fat, 15% from protein and 55% from carbohydrates. The fructose content during the low-fructose diet phases was less than 2% of the caloric intake. During the high-fructose diet phases, the fructose content was set to around 20% of total energy intake. The energy need was individually calculated using the Harris–Benedict-equation 655 + (9.56 × weight (kg) + (1.85 × height (m) − 4.68 × age(years)) × 1.5. A physical activity level of 1.5 was subjected to all patients since none of them reported extreme physical activity or decreased activity below normal. Body weight was determined weekly during the intervention and was also used to check energy balance. The diets and the nutrient intake of the study population have previously been described in more detail [32]. After study inclusion, the subjects underwent the four diet phases (Figure 1).

During the first week, subjects underwent the first low fructose diet phase (lowf1). In this diet phase, subjects had to avoid sweets, highly processed foods, soft drinks and fruits and vegetables containing more than 1g fructose/100 g. A fructose uptake of up to 10 g/day was tolerated. During the second week, subjects followed a high-fructose diet rich in fruits and vegetables (fruit) corresponding to a fructose intake of about 100 g/day. Highly processed foods, sweets and sweetened soft drinks had to be avoided to ensure a low level of free fructose uptake dissolved in beverages or derived from sugar syrups. The third diet week was again a low-fructose diet phase (lowf2) equal to lowf1. The fourth diet phase was a high-fructose diet phase achieved by supplementation with high-fructose syrup (HFS). In this phase, the subjects were asked to sweeten their meals with 100 g/day fructose-glucose-syrup from corn containing 40–44% fructose (C-TruSweet 01750, Cargill Deutschland GmbH, Krefeld). Fructose-rich foods like fruits and juices had to be avoided.

A daily dietary record was conducted by the study subjects to assess the actual nutritional intake and was analyzed using the EBISPro software, version 8.0 (Dr. Erhardt, University of Hohenheim, Stuttgart, Germany). According to these records, study participants varied their energy intake significantly within the different diet phases. During the fruit-rich diet phase, energy intake increased by about 25% and during the HFS diet phase by about 50%. Table 2 shows the energy and nutrient intake of the different diets. The mean nutritional intake of study subjects was described in more detail elsewhere [32]. In brief, during the low-fructose diet phases, subjects consumed higher amounts of fat compared to the defined target. The fruit-rich diet was characterized by a significantly higher intake of fiber compared to the lowf1, lowf2 and HFS diets. To reach a fructose uptake of 100 g/day, high amounts of fruits and vegetables had to be consumed. In fact, sweet fruits like grapes were the preferred choice to reach the defined fructose target of 100 g/day rather than fiber-rich vegetables. During the HFS phase, the energy intake was higher compared to the defined target. This can be explained by the consumption of the fructose-glucose syrup of 100 g/day additionally to the normal diet.

### 2.4. Clinical Study Parameters

Clinical laboratory parameters fasting glucose, alanine aminotransferase (ALT), gamma-glutamyl-transpeptidase (GGT), alkaline phosphatase (AP), high-density lipoprotein (HDL), low-density lipoprotein (LDL), triglycerides (TG), blood sedimentation rate (BSR), creatinine, urea, and uric acid were assessed. Anthropometric and clinical data (body mass index (BMI), waist circumference (WC) and blood pressure) were determined. Liver ultrasound was performed by a trained physician using the LOGIQ P6 device (GE Healthcare, Solingen, Germany) as described [33]. Hepatorenal index (HRI) was determined according to Webb et al. [34], and fatty liver index (FLI) was assessed according to Bedogni et al. [35]. Plasma metabolites were analyzed by targeted metabolomics [32].

### 2.5. Microbiota Analysis by 16S rRNA Amplicon Sequencing

Stool samples were collected weekly after each diet period in a stool sample vessel with DNA stabilizer. The microbial DNA was isolated using the Invitek PSP-Spin Stool DNA Plus Kit with lysis enhancer according to the manufacturer’s instructions (Stratec Molecular, Berlin, Germany). 16S amplicon sequencing was performed by CeMet GmbH, Center for Metagenomics, Tübingen. Twenty-five nanogram of genomic DNA was used to prepare amplicon libraries using Nextera XT Index Kit (Illumina, San Diego, CA, USA), according to manufacturer’s instruction. Primers targeted the V3-V4 region of the 16S rRNA gene [36]. Paired-end sequencing was performed on an Illumina MiSeq platform (IIIumina, San Diego, CA, USA) using v2 reagents. Sequence reads were processed by a bioinformatic pipeline. PRINSEQ-lite was used to trim sequences by base quality [37]. Subsequently quality control of trimmed sequences was performed by FastQC [38]. Fastq-Join was used for merging trimmed sequences [39]. Merged reads with a length shorter 100 bp were filtered and FastQC was applied again. Taxonomic classification of the sequence data was performed against the National Center for Biotechnology Information (NCBI) bacterial 16S rRNA database using MALT [40]. For further analysis, sequence data were analyzed using MEGAN6 software [41] MEGAN6 was used to calculate the bacterial abundance and the Shannon’s diversity index of the volunteers. Normalized read counts were used for further statistical analysis. The functional annotation of the reads was done based on the KEGG library (Kyoto Encyclopedia for Genes and Genomes, http://www.genome.jp/kegg/).

### 2.6. Statistical Analysis

Statistical analysis of 16S rRNA gene amplicon sequencing was performed using R software 3.5.1 (R Core Team, Vienna, Austria) and its packages Hotelling, reshape 2, bindr, ggplot2 and heatmap3 as well as RStudio version 1.1.456 (RStudio, Inc., Boston, MA, USA). GraphPad Prism version 7.0 software (GraphPad Software, Inc., La Jolla, USA) was used for graphical presentation of bacterial abundances as well as for statistical analyses. Laboratory, clinical and nutritional parameters were tested for normal distribution using Kolomogov–Smirnov test. In case of normally distributed data unpaired Student’s *t*-test was used to test for significant differences between group means. Mann–Whitney U test was used to test for differences between group means in case of non-normally distributed data. Mann–Whitney U test was performed for analyzing differences in microbiota abundance between lean and obese subjects. Differences within diet phases were analyzed using paired Student’s *t*-test with Bonferroni correction for multiple testing. Significant changes between phases in microbiota abundance were analyzed using Wilcoxon signed-rank test with Benjamini–Hochberg (false discovery rate (FDR)) correction for multiple comparisons. Pearson’s correlation analysis was performed for analyzing correlations between clinical and anthropometric parameters and microbiome data. *p*-values of < 0.05 were considered as statistically significant.

## 3. Results

### 3.1. Gut Microbiota Profile in Study Subjects

**Figure 2:**
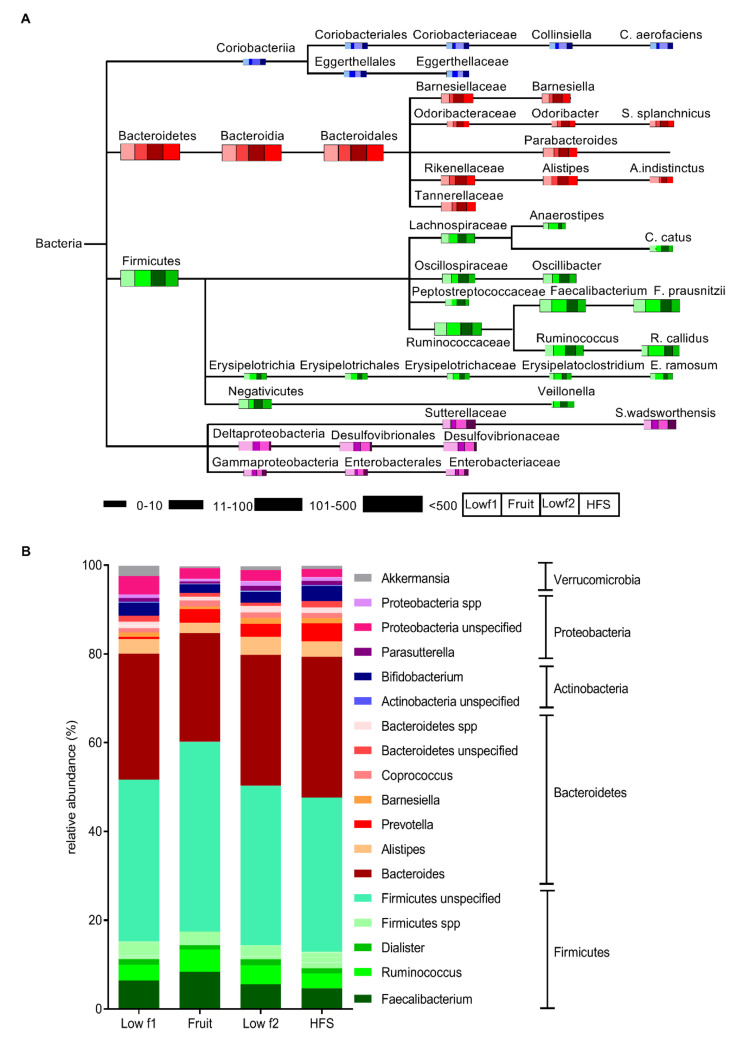
Effects of different fructose diets on the composition of the gut microbiome. (**A**) Composition of bacterial communities that changed among the different fructose diets. The schematic dendogram was constructed from 16S rRNA sequencing analysis results. Size of the bars represents the abundances of the bacterial taxa. Different phyla were shaded by different colors: blue, Actinobacteria; red, Bacteroidetes; green, Firmicutes; purple, Proteobacteria. (**B**) Distribution of the relative abundances at phylum and genus level between the different fructose diets.

The most abundant phyla were *Firmicutes* accounting for 52.6% of abundance at phylum level, *Bacteroidetes* for 36.0%, *Proteobacteria* for 5.7%, *Actinobacteria* for 3.2% and *Verrucomicrobia* for 2.3% (Figure 2B). The remaining phyla accounted for 0.2%. At the genus level *Bacteroides* clearly dominated (28.4%), followed by *Faecalibacterium* (6.4%), *Ruminococcus* (3.6%), *Alistipes* (3.3%) and *Bifidobacterium* (2.9%). During all phases the *Firmicutes* abundance clearly dominated (lowf1, 52.6%; fruit, 61.6%; lowf2, 51.6%; HFS, 48.8%), followed by *Bacteroidetes* (lowf1, 36.0%; fruit, 32.1%; lowf2, 39.9%; HFS, 43.1%), *Proteobacteria* (lowf1, 5.7%; fruit, 3.5%; lowf2, 4.6%; HFS, 3.6%) and *Actinobacteria* (lowf1, 3.2%; fruit, 2.1%.; lowf2, 2.8%; HFS, 3.6%). Among the different diet phases lowf1, fruit, lowf2 or HFS changes in the abundances of bacterial taxa were observed (Figure 2A). The bacterial diversity did not change significantly within the phases, although a minor pattern was marginally visible namely higher indices after fructose-poor diets (lowf1, lowf2) and lower ones after fructose-rich diets (fruits, HFS; data not shown).

### 3.2. Fructose Dependent Changes of Relative Abundance on Phylum Level

**Figure 3:**
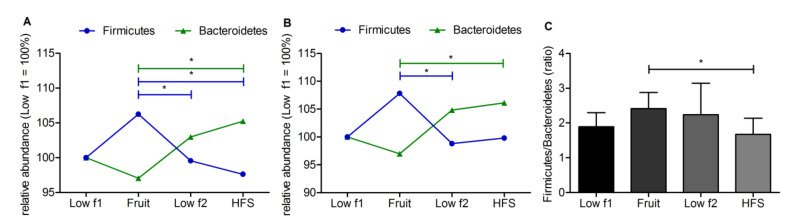
Significant changes between fructose diet phases on phylum level. Relative abundance of Bacteroidetes and Firmicutes in all (*n* = 12) (**A**) and obese (*n* = 6) subjects (**B**). (**C**) Firmicutes/Bacteroidetes ratio of all subjects. Data are shown as mean ± standard error of the mean (SEM). (**A**,**C**) Significant differences are indicated as * adj. *p*-value < 0.05. (**B**) Significant differences are indicated as * *p*-value < 0.05.

At the phylum level, high-fructose diets induced differences in microbiota composition, especially changes in *Firmicutes* and *Bacteroidetes* abundance. Relative abundance of *Firmicutes* was increased after the fruit diet and decreased when study participants switched from the fruit to the lowf2 and HFS diet, whereas the relative abundance of *Bacteroidetes* showed opposing patterns suggesting a fructose-dependent modulation of the gut microbiome (Figure 3). The *Firmicutes* abundance changed significantly from the fruit to lowf2 diet phase (FDR-adjusted *p* = 0.021), whereas changes in *Bacteroidetes* abundance were not significant after FDR correction (*p* = 0.075). Comparing the fruit and HFS diet phases, significant differences were observed in *Firmicutes* and in *Bacteroidetes* abundance (Figure 3A). Analyzing the subgroup of obese subjects separately, a similar pattern in microbiota changes was observed (Figure 3B), although the changes in *Firmicutes* and in *Bacteroidetes* abundance were only significant without correction for multiple testing. The *Firmicutes* to *Bacteroidetes* ratio is regarded to be of significant relevance in human gut microbiota composition. In the *Firmicutes*/*Bacteroidetes* ratio, we observed significant differences between the fructose-rich diets. The ratio of *Firmicutes* to *Bacteroidetes* (F/B ratio) was significantly decreased after the HFS diet compared to the fruit diet (Figure 3C).

### 3.3. Fructose Dependent Changes of Relative Abundance on Phylum Level

**Figure 4:**
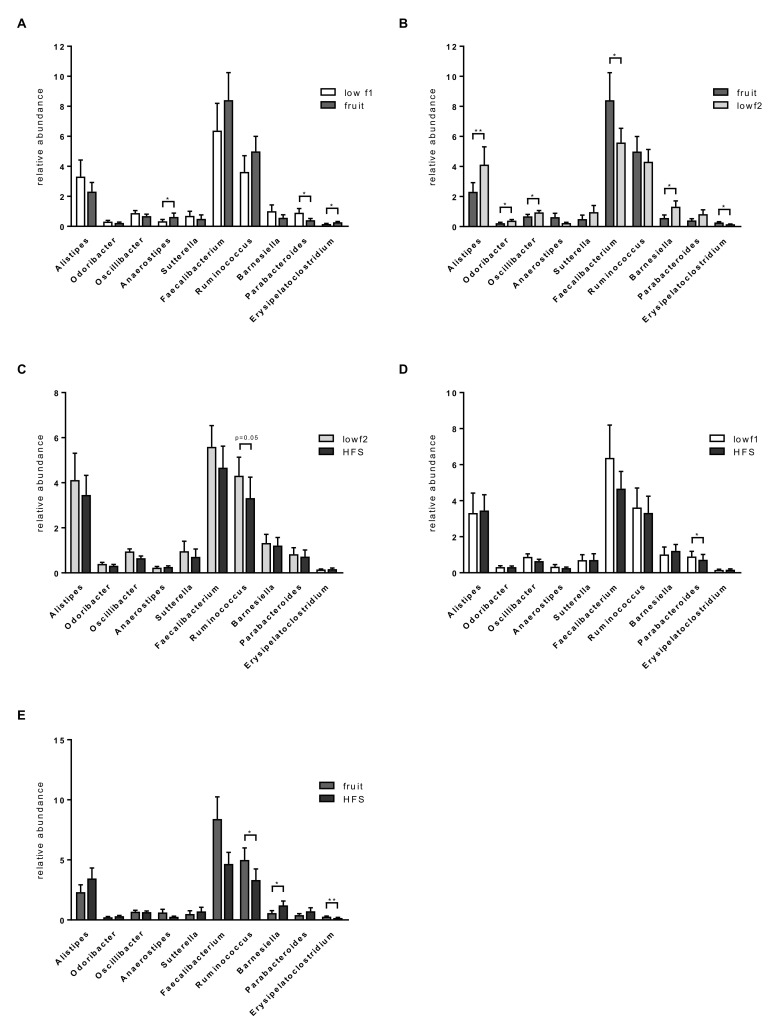
Changes of gut microbiota (relative abundances) on genus level among different fructose diets. (**A**) lowf1 vs. fruit, (**B**) fruit vs. lowf2, (**C**) lowf2 versus high-fructose syrup (HFS), (**D**) lowf1 vs. HFS, (**E**) fruit versus HFS. Significant differences are indicated as * *p*-value < 0.05 and ** *p*-value < 0.01.

Analyzing bacterial abundances on the genus level, we identified ten genera that were significantly different among the diet phases, *Parabacteroides*, *Alistipes*, *Odoribacter*, *Oscillibacter*, *Faecalibacterium*, *Barnesiella*, *Erysipelatoclostridium*, *Ruminococcus, Anaerostipses* and *Veillonella* if no correction for multiple testing was applied. Abundance of the genus *Parabacteroides* was significantly reduced after the fruit diet compared to the low-fructose diet (lowf1), and abundance of *Anaerostipes* was significantly increased (Figure 4A). *Alistipes*, *Oscillibacter*, *Odoribacter* and *Barnesiella* were significantly decreased after the fruit diet compared to the low-fructose diet (lowf2) (Figure 4B). Abundance of the two genera *Faecalibacterium* and *Erysipelatoclostridium* was increased during the fruit diet and significantly decreased between the fruit and the lowf2 phase (Figure 4B). Comparing abundances between the lowf2 and HFS diet, *Ruminococcus* was reduced by the high-fructose syrup diet (*p* = 0.052, Figure 4C). Abundances differed also among the two high-fructose diets, the fruit diet, which is characterized by a high fiber intake, and the HFS diet. The relative abundances of *Ruminococcus* and *Erysipelatoclostridium* were significantly lower after the HFS diet compared to the fruit diet whereas *Barnesiella* abundance was significantly higher (Figure 4E). When comparing abundances between the HFS diet and the lowf1 diet, we found that the abundance of the genus *Veillonella* was significantly higher after the high-fructose diet (data not shown), although the genus was in general of low abundance (<1%). Abundance of *Parabacteroides* significantly decreased after the HFS diet compared to the lowf1 diet (Figure 4D). Microbiota profiles of lean and obese subjects showed similar patterns for most genera during the study phases though these changes were not significant (data not shown). *Ruminococcus* was the only genus the abundance of which was significantly reduced by the high-fructose syrup in the lean subject group but not in the obese subject group (data not shown).

### 3.4. Functional Alterations in the Gut Microbiome during the Response to Fructose

The Kyoto Encyclopedia of Genes and Genomes (KEGG) database analysis revealed a total of 18 biological metabolic pathways involved in the response to fructose, including essential carbohydrate metabolic pathway and amino acid metabolic pathways, fructose and mannose metabolism (K15856), and ascorbate and aldarate metabolism (K13874) (Table S1). The fructose and mannose metabolism pathway were significantly elevated after the fruit diet (adjusted *p* = 0.011, Table S1). The fruit diet also significantly changed the bacterial chemotaxis pathway, the folate biosynthesis pathway and the ABC transporter pathway. In particular, GDP-4-dehydro-6-deoxy-D-mannose reductase (K15856), chemotaxis protein CheX (K03409), molybdenum cofactor cytidylyltransferase (K07141), putative lysine transport system substrate-binding protein (K17073) were all significantly higher after the fruit diet. When analyzing changes between lowf2 and HFS diet, three pathways were affected, the inositol phosphate metabolism, the ascorbate and aldarate metabolism and the two-component system. We found scyllo-inositol 2-dehydrogenase (NAD +) (K16043) significantly more abundant after the HFS diet (adjusted *p* < 0.05, Table S1) and LysR family transcriptional regulator (K18900) and L-arabinonolactonase (K13874) significantly reduced after the HFS diet (adjusted *p* < 0.05, Table S1). The strongest effect of HFS was observed on the inositol phosphate metabolism (adjusted *p* = 6.15 × 10^−22^, Table S1). Comparing the fruit diet with the HFS diet, 14 pathways were significantly changed, including the two-component system, fructose and mannose metabolism, glycerolipid metabolism, pyrimidine metabolism, purine metabolism, oxidative phosphorylation, folate biosynthesis, flagellar assembly, bacterial chemotaxis. From the fructose and mannose metabolism the GDP-4-dehydro-6-deoxy-D-mannose reductase (K15856) significantly decreased (adjusted *p* = 0.046, Table S1).

### 3.5. Correlation Analysis between Microbiota Abundance and Clinical Parameters.

**Figure 5:**
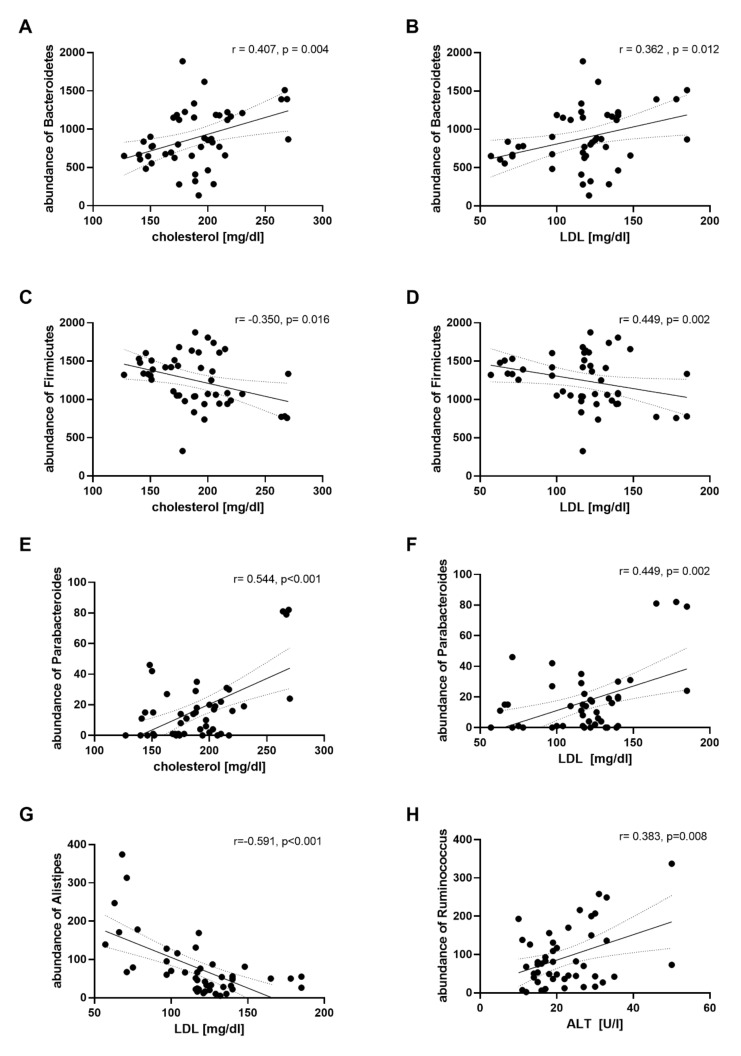
Abundances of specific phyla and genera that correlate with plasma lipid parameters. *Bacteroidetes* abundance vs. cholesterol (**A**) and LDL level (**B**), *Firmicutes* abundance vs. cholesterol (**C**) and LDL level (**D**), *Parabacteroides* abundance vs. cholesterol (**E**) and LDL level (**F**). *Alistipes* abundance vs. LDL level (**G**), *Ruminococcus* abundance vs. ALT level (**H**). Pearson’s correlation analysis is shown. Each dot represents a related pair of values from the study subjects. LDL, low density lipoprotein; ALT, alanine aminotransferase.

To evaluate the association between the clinical parameters and bacterial taxa, a correlation analysis was performed with abundances of all bacterial phyla, families and genera of the study subjects during the four different dietary phases. Correlations within all bacterial taxon and clinical parameters are shown in the supplemental material (Figure S1). Abundance of *Bacteroidetes* positively correlated with plasma cholesterol (*r* = 0.407, *p* = 0.004) and LDL level (*r* = 0.362, *p* = 0.012) (Figure 5A,B). In contrast, abundance of *Firmicutes* was negatively correlated with cholesterol (*r* = −0.350, *p* = 0.016) and LDL level (*r* = −0.292, *p* = 0.047 (Figure 5C,D). On the genus level, abundance of *Parabacteroides* was positively correlated with cholesterol (*r* = 0.544, *p* < 0.001) and LDL level (*r* = 0.449, *p* = 0.002) (Figure 5E,F). Abundance of *Sutterella* was positively correlated with plasma LDL (*r* = 0.390, *p* = 0.007), TG (*r* = 0.574, *p* < 0.001) and cholesterol level (*r* = 0.432, *p* = 0.002) (Figure S1). Plasma levels of LDL were also correlated with the abundance of *Alistipes* (*r* = −0.591, *p* < 0.001) (Figure 5G). Furthermore, a positive correlation between abundance of *Ruminococcus* and levels of ALT was observed (*r* = 0.383, *p* = 0.008) (Figure 5H).

### 3.6. Correlation Analysis between Microbiota Abundance and Plasma Metabolites

**Figure 6:**
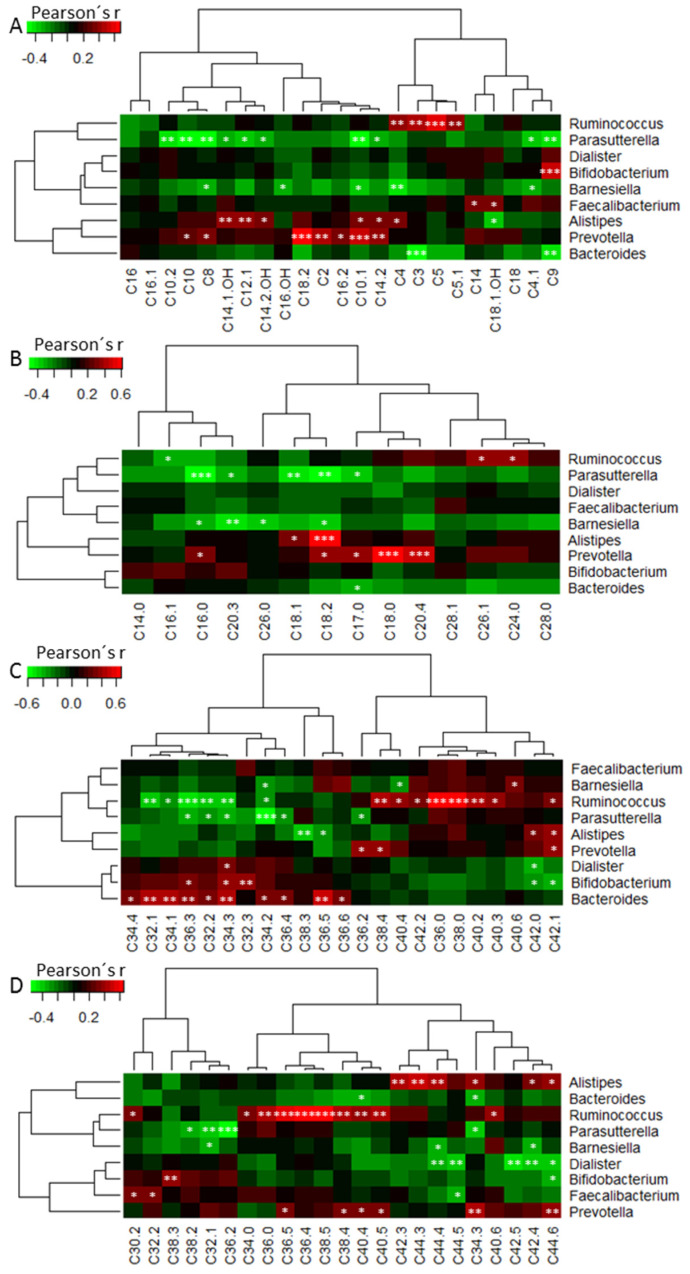
Correlation heatmap (Pearson’s correlation) of most abundant gut microbiota on genus level and plasma metabolites. Microbiota vs. (**A**) acylcarnitines, (**B**) lyso-phosphatidylcholines, (**C**) diacyl-phosphatidylcholines, (**D**) acyl-phosphatidylcholines. Green colored field figure negative and red colored positive correlations. Hierarchical clustering of microbiota abundance and clinical parameter are shown by dendograms.

To focus our analysis further, we correlated the abundance of the genera with plasma metabolites acylcarnitines (AC), lysophosphatidylcholines (lysoPC), diacyl-phosphatidylcholines (PCaa) and acyl-phosphatidylcholines (PCae). The most abundant genus *Faecalibacterium* of the *Firmicutes* correlated positively with two long-chain ACs, whereas the second most abundant one, *Ruminococcus*, showed a positive correlation with short-chain ACs, specifically with AC C4 (Butyryl-L-carnitine) among others (Figure 6A–D). Within the *Bacteroidetes* taxon, mostly negative correlations to ACs were observed for *Bacteroides* (e.g., C3) and *Barnesiella* (e.g., C4) and positive ones for *Alistipes* and *Prevotella* (e.g., C2, Acetyl-L-carnitine). *Parasutterella* abundance (*Proteobacteria*) was negatively correlated with ACs whereas *Bifidobacteria* (Actinobacter) showed a positive correlation to C9 (Figure 6A). A few long-chain lysoPCs correlated in both directions with the abundance of *Ruminococcus*. Within the *Bacteroidetes* taxon, prevailing positive (*Alistipes* and *Prevotella*) but also negative (*Barnesiella*) correlations were found. *Parasutterella* (*Proteobacteria*) correlated negatively to some lysoPCs (Figure 6B). Within the Firmicutes taxon, PCaas correlated in both directions largely equilibrated whereby *Bacteroides* (*Bacteroidetes*) were found to correlate mostly positively (Figure 6C). Bacteria from the *Firmicutes* and *Bacteroidetes* taxon were found to correlate with PCaes approximately equilibrated in both directions (Figure 6D).

## 4. Discussion

In the present study, we demonstrate that diets differing in the amount of the fructose content and the source of the fructose mediate alterations of the gut microbiota differently in healthy humans. Abundance of the phyla *Firmicutes* and *Bacteroidetes*, which represent the largest proportion of the gut microbiota, changed in response to the different high-fructose diets. The relative abundance of *Firmicutes* increased after a fruit diet and decreased following the HFS diet, whereas the relative abundance of *Bacteroidetes* showed opposing patterns. At the genus level, we observed more specific shifts including an increase in *Faecalibacterium* and *Anareostipes* and a reduction in *Parabacteroides* and *Barnesiella* after the fruit-rich diet. After the high-fructose syrup diet (HFS), we observed a decrease of *Ruminococcus*, *Faecalibacterium* and *Erysipelatoclostridium* whereas *Barnesiella* abundance was higher after the HFS diet.

High consumption of fructose, one of the critical risk factors contributing to the development of NAFLD, increases gut permeability and alters the microbiota composition in the gastrointestinal tract and therefore promotes bacterial translocation and metabolic endotoxemia resulting in lipid accumulation and low-grade inflammation leading finally to hepatic steatosis and metabolic disease. Low doses of fructose are cleared by the small intestine, but high doses of fructose saturate the absorption and catabolism of fructose leading to fructose spill-over not only to the liver [42] but also to the colonic microbiota, which further metabolizes fructose [43]. The resulting metabolites become an energy source for bacteria and influence the gut environment [44]. Here we report, for the first time, that the human intestinal microbiota is altered differently in response to dietary fructose in different concentrations and sources, which is uncovered by significant taxonomic differences between the different fructose diet phases.

Our finding of an increased *Firmicutes* and a reduced *Bacteroidetes* abundance in healthy adults after a high-fructose diet derived from fruit and vegetables confirms and extents previous findings. The first evidence that fructose might affect microbiota arose from studies in rodent models [28,45]. Fructose-fed mice showed a significantly lower abundance of *Bacteroidetes* and a slightly increased *Firmicutes* abundance [26]. The microbiota changes we observed at the phylum level are consistent with these data and also in agreement with our own previous findings that a high-fructose diet caused a reduced *Bacteroidetes* abundance and a slightly enhanced *Firmicutes* abundance [6]. Furthermore, our data are in accordance with a study by Astbury et al. [46], who found a higher *Firmicutes* and a lower *Bacteroides* abundance after a high-fructose diet during pregnancy in rats. However, it should be noted that all these studies analyzed the effects of fructose either added to the solid diet or as an additive in drinking water, but not as fructose from whole fruits. As a result of a lower *Bacteroidetes* and a higher *Firmicutes* abundance, the *Firmicutes* to *Bacteroidetes* (*F/B*) ratio was slightly increased after the fruit diet. This is in contrast to previous literature which proposed the *F/B* ratio to be a contributing factor to obesity and its relative metabolic disease although results in humans are controversial. Several studies did not observe any changes in the *F/B* ratio or even reported a decreased ratio in obese human individuals [47,48,49]. Others reported an increased *F/B* ratio only in obese with metabolic alterations [50]. The discrepancies may relate to methodological differences between studies, to population differences (obese individuals with or without metabolic disease) or to dietary influences.

Besides a high fructose content, the fruit diet was characterized by a relatively high content of fiber, and study subjects had a significantly higher fiber intake during the fruit phase than during the HFS diet. The observed alterations in gut microbiota may at least partially reflect changes driven by a higher intake of fiber rather than changes in response to the high-fructose consumption. Our results are in line with the results of Wu et al. who reported that abundance of *Firmicutes* was positively associated with a high-fiber diet, whereas *Bacteroidetes* abundance was predominantly negatively associated with fiber intake [51]. *Firmicutes* abundance was also increased in humans following a short-term dietary intervention characterized by a high-fiber content, and this was accompanied by a decreased abundance of several genera from the *Bacteroidetes* phylum [52]. Similarly, other studies described a decreased abundance of the *Bacteroidetes* taxon after a fiber rich diet, e.g., of the class *Bacteroidia* [53] and genus *Bacteroides* [54]. Consistent with these data, we observed a lower abundance of *Bacteroidetes* after the fruit diet. Abundance of *Barnesiella*, a common gut bacterium present in healthy individuals and genus of the order *Bacteroidales*, was also significantly reduced after the fruit diet compared to lowf2 diet phase.

Dietary fibers from fruit and vegetable are degraded by colonic bacteria resulting in the production of key metabolites such as short chain fatty acids (SCFAs), which are important promoters of gut health. SCFAs, particularly butyrate, are well known for their anti-inflammatory functions [55], antimicrobial activity and maintenance of intestinal barrier function and may be protective against several diseases, including diabetes and obesity [56]. Interestingly, in our study, a high intake of fructose from fruit and vegetables increased abundance of the genera *Anaerostipes*, *Coprococcus*, *Ruminococcus* (though effects for *Coprococcus* and *Ruminococcus* were not significant) and *Erysipelatoclostridium* all belonging to butyrate-producing bacteria of the phylum *Firmicutes*. The two families *Lachnospiraceae* and *Ruminococcacae* include most of the known butyrate producers [57,58]. The significantly increased abundance of *Anaerostipes* (family *Lachnospiraceae*) after the fruit diet is supported by previous findings describing the ability to metabolize fructose [59,60]. Furthermore, our results are in line with a previous study showing that a fructose-supplemented diet increased the abundance of *Anaerostipes* genus [61]. The high-fructose fruit diet also enriched abundance of *Coprococcus* (family *Lachnospiraceae*) and *Ruminococcus* (family *Ruminococcacae*). Interestingly, the abundance of *Ruminococcus* was positively associated with short-chain ACs, among others with AC C4 (Butyryl-L-carnitine) suggesting that butyrate delivered by *Ruminococcus* might be responsible for the elevated blood AC C4:1 level after the fruit diet. Similar changes in the gut microbiota have been observed in studies in which the abundance of the genera *Coprococcus* and *Ruminococcus* was increased by a fructose-rich diet in mice [28,29,62,63]. One of the most abundant butyrate producers in the human gut is *Faecalibacterium prausnitzi,* which also ferments fructose [64]. Supporting the hypothesis that a fructose and fiber-rich diet shifts microbiota towards butyrate producers we found the genus *Faecalibacterium* significantly increased after the fruit diet compared to the low-fructose diet. In summary, a high-fructose fruit diet led to an increase of butyrate-producing bacteria, which may promote a healthy gut function.

A clear increase in the consumption of fructose has occurred in recent decades particularly in form of HFS, which is used as sweetener in soft drinks and other sweetened beverages and in processed foods [12]. Western-style diets high in HFS not only increase the risk of NAFLD but also contribute to the rise and prevalence of obesity [65]. Most recently, the demonstration that dietary fructose stimulates hepatic lipogenesis via microbiota-derived acetate determined a previously unappreciated interaction among diet, the gut microbiome and the host metabolism that contributes to fructose-induced NAFLD [66]. In the current study, we found that the gut microbiota composition was distinctively different after a short-term HFS diet compared to after the fruit-rich diet. This difference might be related to different absorption rates for fructose in the small intestine depending on the fruit matrix or the fiber content leading to different outcomes in microbiota metabolism. The observation of an increased abundance of *Bacteroidetes* after the HFS diet aligns with a previously reported increase in *Bacteroides* after high-fructose syrup liquid diet in mice [62] and an increased abundance of *Bacteroidetes* in rats fed a high-fructose diet [30]. *Bacteroidetes* bacteria, which compromise the largest phylum of Gram-negative bacteria in the gut microbiome, are the most abundant contributors to LPS biosynthesis [67]. A high abundance of *Bacteroidetes* has been associated with liver inflammation [25,68,69] and the development of NAFLD and further metabolic diseases [70]. The correlation between the *Bacteroidetes* abundance and blood cholesterol and LDL levels supports the hypothesis that a higher *Bacteroidetes* abundance induced by the HFS diet may be associated with increased blood lipid levels. A decreased abundance of *Firmicutes* after the HFS diet, which was negatively correlated with blood cholesterol and LDL levels, matches these findings and further supports the assumption that HFS diet-induced microbiota changes may have unfavorable health effects.

Interestingly, within the phylum *Firmicutes*, abundance of *Erysipelatoclostridium* and *Ruminococcus* was reduced after the HFS diet compared to the fruit diet, whereas abundance of *Veillonella* was increased after the HFS compared to the lowf1 diet. Some *Veillonella* species are able to ferment fructose and fructose can be incorporated into the *Veillonella* LPS and thus may be of major importance in the production of LPS [71]. We therefore hypothesize that an increased *Veillonella* abundance induced by the HFS diet could contribute to the production of endotoxins and increase LPS level. Our results are in line with a recent study by Li et al. [72] who found the genus *Veillonella* to have increased after a high-fructose diet in mice.

In contrast to studies in rodent models reporting an increased *Ruminococcus* abundance after a high-fructose diet [6,28,29], *Ruminococcus* abundance was not significantly changed following the HFS diet compared to low fructose diet. These differences might be at least partially explained by different diet formulations as in the animal experiments fructose was incorporated in the solid diet whereas our study analyzed the effect of high-fructose corn syrup. In addition, caution should be taken in interpreting studies of the fructose effects in animal experiments because results from animal models cannot always be translated to humans. Compared to the high-fructose fruit diet *Ruminococcus* was of significantly lower abundance after the HFS diet hinting at a dietary fiber-related effect. Analyzing the lean study group separately, we found *Ruminococcus* being significantly reduced by the HFS diet compared to the lowf2 diet. As study subjects had a similar intake of fiber and fat during the lowf2 and the HFS diet phases, this effect cannot be attributed to differences in the consumption of dietary fiber. Bacteria of the genus *Ruminococcus* are key players in the dynamics of gut microbial communities and have been associated with gastrointestinal health benefits in humans [73]. In summary, our results point to a reduction of the beneficial butyrate producing *Ruminococcus* bacteria after the HFS diet. Furthermore, our data suggest that the HFS syrup containing a mix of fructose and glucose as monosaccharides leads to a different outcome in microbiota metabolism compared to dietary fructose from fruit and vegetable where it is also found bonded to glucose as the disaccharide sucrose and thus affects microbiota composition and *Ruminococcus* abundance differently. Besides this, intake of dietary glucose during the HFS diet was significantly higher than during the fruit diet (data not shown), which could also play a role in different effects of the two high-fructose diets. Furthermore, fructose from HFS in contrast to fructose from fruit and vegetables, which is absorbed and released consistently from the food matrix in smaller amounts, may persist for a longer time within the gut and, thus, be metabolized by enterocytes and potentially by microbiota of the large intestine.

Remarkably, abundance of *Parabacteroides*, known for its anti-obesity effects, was significantly decreased by both high-fructose diets compared to the lowf1 diet though the HFS diet-induced decrease was less pronounced. These results are consistent with findings from our own group demonstrating that *Parabacteroides* abundance was reduced after a high-fructose diet [6]. Our observations align with a previous report in which *Parabacteroides* species were exclusively observed in rats fed a control diet and not in a fructose-rich diet [46]. Recently, the gut commensal *Parabacteroides distasonis* was shown to alleviate obesity [74] further strengthening the assumption that a high-fructose diet hampers health promoting microbiota and might thus promote metabolic diseases.

A potential limitation of this study is the small sample size, which, together with a high inter-individual variability, may have affected the statistical power. Differences in the baseline microbial composition can drive the response to dietary changes [75,76], which is highly individualized [77,78]. Interindividual differences in the response to dietary fructose might therefore have weakened mean microbiota changes between different diet groups. Furthermore, one-week dietary phases might have led us to miss potential long-term effects on microbiota. Though rearrangements in the human microbiome have been observed 24 h after initiating a diet [47,79], most of the studies analyzed short-term effects on microbiota composition after two weeks of dietary intervention. While a significant decrease of gut community diversity was found after two weeks of short-term diet [48], no significant differences in α-diversity were detected after a short-term diet of 5 days [80]. This is in line with our observation that α-diversity did not change significantly between the one-week diet phases. Because of the short-term intervention phases, carry-over effects, namely, during the low f2 phase, cannot be excluded. This might explain some differences we found between the low f1 and low f2 phases despite of the fact that the diet was the same in these two phases.

## 5. Conclusions

In conclusion, our results demonstrate that short-term high-fructose diets differing in both amount and source of fructose mediate alterations of the gut microbiota composition differently. Importantly, we provide evidence that the HFS diet induces an imbalanced microbiota profile characterized by a significantly reduced abundance of beneficial butyrate-producing bacteria and of bacteria known for anti-obesity effects which could cause disruption of mucosal homeostasis and gut function.

Despite the high-fructose content, the fruit-rich diet shifts the intestinal microbiota composition towards a healthy butyrate-producing community, suggesting that effects which may be attributed to fiber content of fruit and vegetable prevail and influence the microbiota composition in an protective manner counteracting the harmful effects of excessive fructose. However, future studies are needed to further investigate the mechanism of action by which fructose elicits its versatile functions on host microbiota and the role of fructose-mediated changes in the gut microbiota.

## Figures and Tables

**Table 1:** Baseline characteristics of the study population.

Characteristics	Lean (*n* = 6)	Obese (*n* = 6)	*p*-Value
Age (years)	26 ± 2	30 ± 3	0.013
BMI (kg/m^2^)	22.5 ± 1.5	41.5 ± 4.0	0.004
WC (cm)	72.4 ± 2.5	118.3 ± 9.4	0.004
BP sys (mmHg)	104.2 ± 7.4	122.5 ± 9.9	0.004
BP dias (mmHg)	69.2 ± 6.7	83.3 ± 6.8	0.005
FBG (mg/dL)	87.7 ± 4.6	93.3 ± 8.4	ns
HDL (mg/dL)	62.0 ± 11.4	53.8 ± 14.2	ns
LDL (mg/dL)	100.0 ± 22.1	137.2 ± 35.4	0.025
TG (mg/dL)	82.0 ± 20.5	108.7 ± 63.7	ns
FLI	7.0 ± 2.5	89.8 ± 12.5	0.001
HRI	0.9 ± 0.2	1.0 ± 0.1	ns
GGT	15.5 ± 2.2	25.5 ± 17.7	ns
ALT	16.2 ± 3.1	23.7 ± 6.2	0.024

Data are expressed as mean (± SD). Abbreviations: ALT, alanine aminotransferase; BMI, body mass index; BP sys, systolic blood pressure; BP dias, diastolic blood pressure; FBG, fasting blood glucose; FLI, fatty liver index; GGT, gamma-glutamyl-transpeptidase; HDL, high-density lipoproteins; HRI, hepatorenal index; LDL, low-density lipoprotein; NW, normal weight; OB, obese; TG, triglycerides; WC, waist circumference.

**Table 2:** Composition of study diets.

Nutrients	Low Fructose Diet		Fruits		HFS	
	Goal	Actual Intake	Goal	Actual Intake	Goal	Actual Intake
Energy (kcal/day)						
NW	2002 ± 56	1828 ± 260	2005 ± 54	2188 ± 357	2006 ± 59	2404 ± 404 *
OB	2207 ± 51	1949 ± 442	2209 ± 51	2404 ± 410	2210 ± 51	2961 ± 273 *
Protein (g/day)						
NW	73 ± 2	83 ± 10	73 ± 2	69 ± 15	73 ± 2	65 ± 10
OB	81 ± 2	102 ± 41 *	81 ± 2	91 ± 29	81 ± 2	92 ± 28
Fat (g/day)						
NW	65 ± 2	80 ± 10 *	65 ± 2	67 ± 20	65 ± 2	70 ± 20
OB	71 ± 2	89 ± 25 *	71 ± 2	79 ± 24	71 ± 2	93 ± 18
CHO (g/day)						
NW	268 ± 8	176 ± 52 *	271 ± 5	308 ± 45	271 ± 5	357 ± 58
OB	296 ± 7	174 ± 24 *	288 ± 4	314 ± 24	288 ± 4	416 ± 47
Fiber (g/day)						
NW	medium	17 ± 6	Medium	36 ± 8	low	12 ± 5
OB	medium	19 ± 8	Medium	39 ± 7	low	19 ± 7
Complex CHO (g/day)						
NW	258 ± 8	160 ± 48 *	171 ± 5	128 ± 5 *	171 ± 5	121 ± 30 *
OB	286 ± 7	166 ± 19 *	188 ± 4	131 ± 3 *	188 ± 4	174 ± 32
Fru (g/day)						
NW	10	7 ± 2	100	100 ± 12	100	106 ± 11
OB	10	4 ± 3	100	102 ± 4	100	108 ± 6
Glu (g/day)						
NW	low	7 ± 2	Medium	80 ± 8	High	130 ± 12
OB	low	5 ± 3	Medium	80 ± 9	High	134 ± 6

Data are expressed as mean (± SD). * More than 20% difference between the proposed amount and the actual intake. Abbreviations: CHO, carbohydrates; Fru, fructose; Glu, glucose; HFS, high-fructose syrup; NW, normal weight; OB, obese; actual intakes of fructose and glucose calculated include the amounts of fructose and glucose bound in sucrose.

## Supplementary Materials

The following are available online at https://www.mdpi.com/2072-6643/12/11/3444/s1. Figure S1: Correlation heatmap (Pearson’s correlation) of gut microbiota within 6 taxon levels and clinical parameters. Table S1: KEGG modules significantly associated with diet.
