# Supplementary material for: Fructose-Induced Intestinal Microbiota Shift Following Two Types of Short-Term High-Fructose Dietary Phases
Source: Nutrients. 2020 Nov 10;12(11):3444. doi: 10.3390/nu12113444 (PMC7697676; doi:10.3390/nu12113444)
Supplement: Supplementary file 1 [file nutrients-12-03444-s001.pdf]

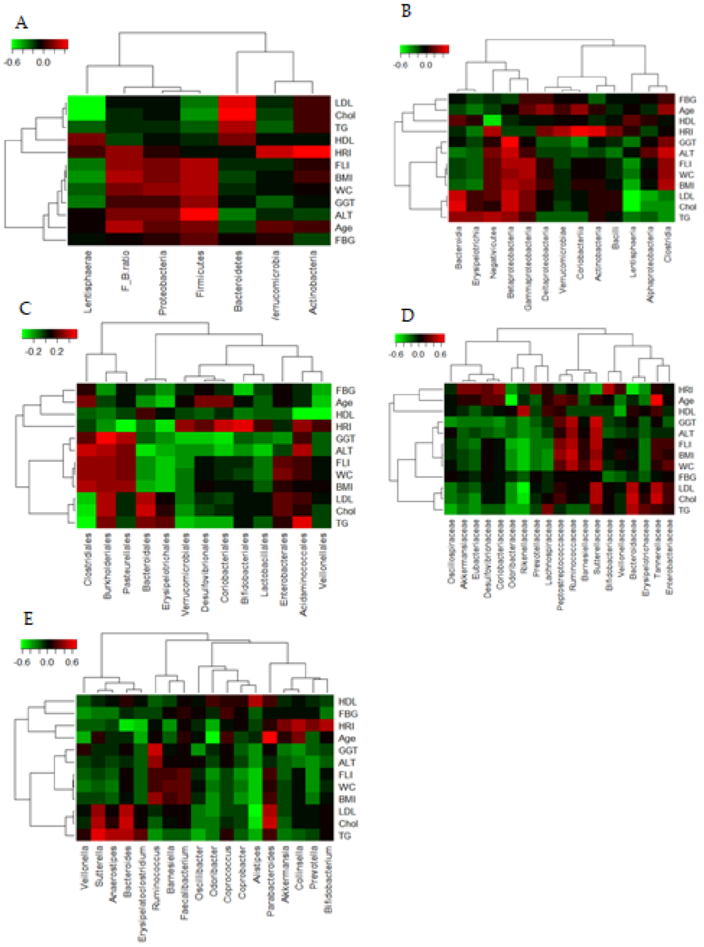

Figure S1. Correlation heatmap (Pearson´s correlation) of gut microbiota within 6 taxon levels and clinical parameters. (**A**) Phyla, (**B**) Class, (**C**) Order, (**D**) Family, (**E**) Genus. Green colored field figure negative and red colored positive correlations. Hierarchical clustering of microbiota abundance and clinical parameter is shown by dendograms. Abbreviations: ALT, Alanine Aminotransferease; BMI, body mass index; Chol, total blood cholesterol; FBG, fasting blood glucose; FLI, fatty liver index; GGT, gamma-glutamyl-transpeptidase; HDL, high density lipoprotein; HRI, hepatorenal index; LDL, low density lipoprotein; TG, triglycerides; WC, waist circumference. Pearson`s r.

Table S2. KEGG modules significantly associated with diet.

Diet phase	KEGG no	Annotation	*p*-value adj.
lowf1 vs fruit	K15856	GDP-4-dehydro-6-deoxy-D-mannose reductase	0,01144670
	K19075	CRISPR-associated protein Cst2	0,01144670
	K19090	CRISPR-associated protein Cas5t	0,01144670
	K03409	chemotaxis protein CheX	0,01727021
	K07141	molybdenum cofactor cytidylyltransferase	0,03339624
	K17073	putative lysine transport system substrate-binding protein	0,03949571
fruit vs lowf2	K16043	scyllo-inositol 2-dehydrogenase (NAD+)	9,3400E-21
	K13874	L-arabinonolactonase	1,1628E-04
lowf2 vs HFS	K16043	scyllo-inositol 2-dehydrogenase (NAD+)	6,1546E-22
	K18900	LysR family transcriptional regulator, regulator for bpeEF and oprC	1,2372E-06
	K13874	L-arabinonolactonase	3,3376E-05
fruit vs HFS	K00567	methylated-DNA-[protein]-cysteine S-methyltransferase	0,04642214
	K00756	pyrimidine-nucleoside phosphorylase	0,04642214
	K01602	ribulose-bisphosphate carboxylase small chain	0,04642214
	K03608	cell division topological specificity factor	0,04642214
	K05356	all-trans-nonaprenyl-diphosphate synthase	0,04642214
	K05573	NAD(P)H-quinone oxidoreductase subunit 2	0,04642214
	K07141	molybdenum cofactor cytidylyltransferase	0,04642214
	K07769	two-component system, OmpR family, sensor histidine kinase NblS	0,04642214
	K07816	putative GTP pyrophosphokinase	0,04642214
	K15856	GDP-4-dehydro-6-deoxy-D-mannose reductase	0,04642214
	K18814	putative inorganic carbon (hco3(-)) transporter	0,04642214
	K19003	1,2-diacylglycerol 3-beta-glucosyltransferase	0,04642214
	K00464	all-trans-8'-apo-beta-carotenal 15,15'-oxygenase	0,04737248
	K00786	glycosyltransferase	0,04737248
	K02293	15-cis-phytoene desaturase	0,04737248
	K02294	beta-carotene hydroxylase	0,04737248
	K02396	flagellar hook-associated protein 1 FlgK	0,04737248
	K02398	negative regulator of flagellin synthesis FlgM	0,04737248
	K02407	flagellar hook-associated protein 2	0,04737248
	K02411	flagellar assembly protein FliH	0,04737248
	K02418	flagellar protein FliO/FliZ	0,04737248
	K02634	apocytochrome f	0,04737248
	K02637	cytochrome b6-f complex subunit 4	0,04737248
	K02638	plastocyanin	0,04737248
	K02640	cytochrome b6-f complex subunit 5	0,04737248
	K02691	photosystem I subunit VII	0,04737248
	K02693	photosystem I subunit IV	0,04737248
	K02694	photosystem I subunit III	0,04737248
	K02697	photosystem I subunit IX	0,04737248
	K02704	photosystem II CP47 chlorophyll apoprotein	0,04737248
	K02707	photosystem II cytochrome b559 subunit alpha	0,04737248
	K02709	photosystem II PsbH protein	0,04737248
	K02711	photosystem II PsbJ protein	0,04737248
	K02716	photosystem II oxygen-evolving enhancer protein 1	0,04737248
	K02717	photosystem II oxygen-evolving enhancer protein 2	0,04737248
	K02722	photosystem II PsbX protein	0,04737248
	K02723	photosystem II PsbY protein	0,04737248
	K02724	photosystem II PsbZ protein	0,04737248
	K03406	methyl-accepting chemotaxis protein	0,04737248
	K03487	LacI family transcriptional regulator, asc operon repressor	0,04737248
	K03610	septum site-determining protein MinC	0,04737248
	K04061	flagellar biosynthesis protein	0,04737248

K05371	phycocyanobilin:ferredoxin oxidoreductase	0,04737248
K05572	NAD(P)H-quinone oxidoreductase subunit 1	0,04737248
K05578	NAD(P)H-quinone oxidoreductase subunit 6	0,04737248
K05579	NAD(P)H-quinone oxidoreductase subunit H	0,04737248
K05580	NAD(P)H-quinone oxidoreductase subunit I	0,04737248
K05581	NAD(P)H-quinone oxidoreductase subunit J	0,04737248
K05582	NAD(P)H-quinone oxidoreductase subunit K	0,04737248
K05583	NAD(P)H-quinone oxidoreductase subunit L	0,04737248
K05584	NAD(P)H-quinone oxidoreductase subunit M	0,04737248
K05585	NAD(P)H-quinone oxidoreductase subunit N	0,04737248
K07777	two-component system, NarL family, sensor histidine kinase DegS	0,04737248
K07813	accessory gene regulator B	0,04737248
K07978	GntR family transcriptional regulator	0,04737248
K08384	stage V sporulation protein D (sporulation-specific penicillin-binding protein)	0,04737248
K08902	photosystem II Psb27 protein	0,04737248
K08903	photosystem II 13kDa protein	0,04737248
K14330	fatty aldehyde-generating acyl-ACP reductase	0,04737248
K14331	fatty aldehyde decarbonylase	0,04737248
K15226	arogenate dehydrogenase (NADP+)	0,04737248
K17734	serine protease AprX	0,04737248
K18475	lysine-N-methylase	0,04737248
K18534	MPBQ/MSBQ methyltransferase	0,04737248
K18640	plasmid segregation protein ParM	0,04737248
K19075	CRISPR-associated protein Cst2	0,04737248
K19090	CRISPR-associated protein Cas5t	0,04737248
K02413	flagellar FliJ protein	0,04977591
